# Using Multidisciplinary Focus Groups to Inform the Development of mI SMART: A Nurse-Led Technology Intervention for Multiple Chronic Conditions

**DOI:** 10.1155/2016/7416728

**Published:** 2016-07-18

**Authors:** Jennifer A. Mallow, Laurie A. Theeke, Elliott Theeke, Brian K. Mallow

**Affiliations:** ^1^School of Nursing, West Virginia University, 9620 HSC South, Morgantown, WV 26506, USA; ^2^Sovern Run, LLC, 210 Cardinal Lane, Albright, WV 26519, USA

## Abstract

Used as integrated tools, technology may improve the ability of healthcare providers to improve access and outcomes of care. Little is known about healthcare teams' preferences in using such technology. This paper reports the findings from focus groups aimed at evaluating a newly developed primary care technology platform. Focus groups were completed in academic, outpatient, and community settings. Focus groups were attended by 37 individuals. The participants included professionals from multiple disciplines. Both prescribing (*N* = 8) and nonprescribing healthcare team members (*n* = 21) completed the focus groups and survey. The majority were practicing for more than 20 years (44.8%) in an outpatient clinic (62%) for 20–40 hours per week (37.9%). Providers identified perceived obstacles of patient use as ability, willingness, and time. System obstacles were identified as lack of integration, lack of reimbursement, and cost. The positive attributes of the developed system were capability for virtual visits, readability, connectivity, user-friendliness, ability to capture biophysical measures, enhanced patient access, and incorporation of multiple technologies. Providers suggested increasing capability for biophysical and symptom monitoring for more common chronic conditions. Technology interventions have the potential to improve access and outcomes but will not be successful without the input of users.

## 1. Background 

Technology is beginning to permeate health care and is being used in multiple ways including helping patients seek out health information via the web, scheduling appointments, refilling medications, sending and receiving secure messages, managing chronic conditions, keeping personal health records, performing self-management, using remote monitoring devices, and building social networks based on similar health concerns [1]. If technology interventions are integrated into existing delivery systems, the potential exists to increase access to care and subsequently improve the health of individuals and populations. Though the use of individual technologies has been effective in improving patient outcomes, this knowledge has not translated into the development of an integrated system to enhance the delivery of primary care. Technology is developing in piecemeal and most applications use one methodology, treat or monitor one illness, and are incompatible with one another or existing Electronic Health Records (EHR) [2]. In addition, evidence is lacking about provider input into the early stages of software development. Provider input from the beginning stages of development of emerging technologies would give opportunities to build, shape, and apply the technology [3]. Thus, this input would potentially enhance future sustainability and scalability in real world delivery systems.

A new platform that integrates multiple technologies for primary health care called mI SMART (Mobile Improvement of Self-Management Ability through Rural Technology) is developed and is being evaluated for feasibility in persons with chronic conditions. The integrated technologies of mI SMART combine a HIPAA compliant, web-based, system of mHealth sensors, and mobile devices to treat and monitor multiple chronic conditions. The mI SMART system allows patients to track diagnoses, medications, and lab results, receive reminders for self-management, perform self-monitoring, obtain feedback in real time, engage in education, and attend e-visits (video conferencing). The system displays a record database to patients and providers that will be integrated into existing Electronic Health Records. The study protocol, development, and feasibility of the mI SMART are published and available for review [4–6]. The creation of mI SMART was guided by the model for developing complex nursing interventions [7].

The model suggests a process for building and informing interventions with the intention of making interventions effective, sustainable, and scalable. Each step in the model builds from and informs the previous step. The steps are as follows: problem identification, practice analysis, identification of the overall objective of the intervention, identification of theory or key principles to guide the intervention, building the intervention and planning the delivery of the intervention, modelling the intervention and seeking expert review, and developing the study protocol [7]. This paper presents the findings related to “seeking expert review” which occurred in the form of provider focus groups. We sought information to inform the development and implementation of mI SMART to improve understanding of providers' needs when using technology to improve health. We assessed preferences and perceived obstacles of multidisciplinary healthcare team members through 3 activities: (1) demonstration of the mI SMART platform, (2) group discussion, and (3) confidential questionnaire. The purpose of this paper is to report the findings of the focus groups which were used to revise mI SMART prior to feasibility testing.

## 2. Methods

Focus group methodology as described by Krueger and Casey [8] was used. A focus group is an interview technique that uses purposive sampling to select participants, who are of a specific population, share similar characteristics, and have something to say about the topic [9]. One of the distinct features of focus group interviews is group dynamics. The type and range of data generated through the interaction of the group members are often deeper and richer than those obtained from a one-on-one interview [9].

### 2.1. Subjects and Site Selection

Focus groups were completed in three different populations including academic healthcare providers (group 1), interprofessional outpatient healthcare providers (group 2), and community stakeholders (group 3). The setting was in north central West Virginia, the location where mI SMART will be implemented. These populations were selected so that the population of future users of the mI SMART platform would be able to participate in the focus groups. Each focus group was held after a regularly scheduled meeting of the group, was arranged with the assistance of the organizer, and was announced a month prior to the planned focus group. In addition, flyers were hung to remind potential participants of the focus groups. A total of 37 participants attended one of the three demonstrations of the mI SMART platform. Of those who attended the demonstration, 31 participated in the group discussion and 29 participants answered the confidential questionnaire. Focus group participants included Medical Assistants, Registered Nurses, front desk staff, Physicians, Nurse Practitioners, Physician's Assistants, Pharmacists, Social Workers, Administrators, and Community Board Members.

### 2.2. Demonstration of mI SMART

The demonstration of mI SMART was done through a combination of PowerPoint slides with screen shots, displaying the platform, and demonstrating the actual equipment to be used. Participants were also able to try the equipment and platform by using a tablet and Bluetooth enabled self-monitoring devices including a glucometer, blood pressure cuff, and weight scale. The demonstration of mI SMART system included a mock e-visit, viewing examples of planed distance education materials, and viewing a mock patient record which included current prescriptions with a links to patient education, a list of current diagnosis with links to patient education, a database of self-monitoring results color coded to normal, above normal, and critical, automated responses for self-monitoring results, patient reminders/notifications, lab results, survey collection links, and short education videos related to common chronic illness control.

### 2.3. Focus Group Process

The focus groups were planned to be 60 minutes long. To provide consistency, the same person, JM, facilitated all three groups. Attendance at focus groups was voluntary. Directly following the demonstration, open ended questions were posed to each group to elicit responses and conversation from the healthcare team members. The following open ended questions were asked to each group:

- What do you think your biggest obstacle is when thinking about prescribing mHealth tools?
- As a patient yourself, what do you think your biggest obstacle is when thinking about using mHealth tools?
- What did you like best about the mI SMART platform?
- What would you like to see removed from the mI SMART platform?
- What would you like to see added to the mI SMART platform?
- Which outcome measures do you think would be most helpful to measure when treating patients with chronic illness using technology?
- What outcome measures do you think are most important to patients?
- What else would you like to tell me about mI SMART?

**Figure 1:**
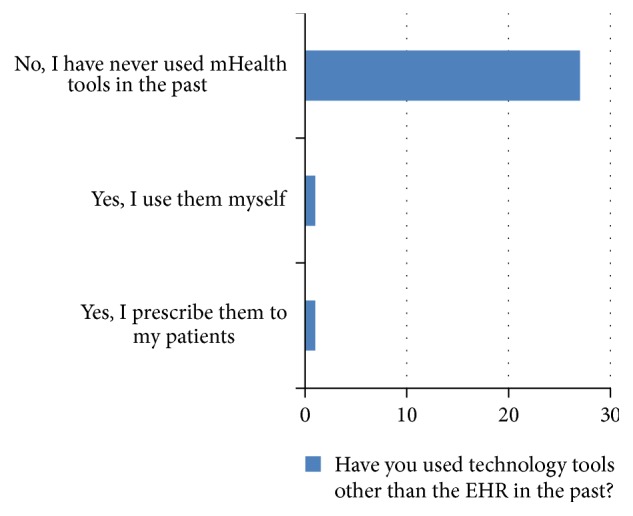
Past use of technology.

**Figure 2:**
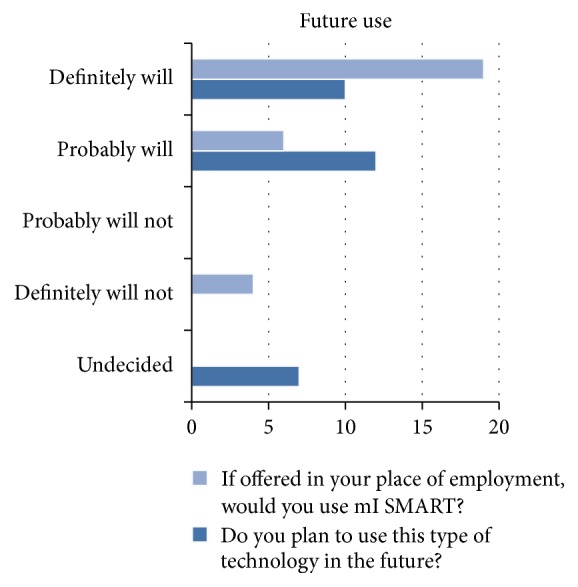
Future use of mI SMART and other technologies.

**Figure 3:**
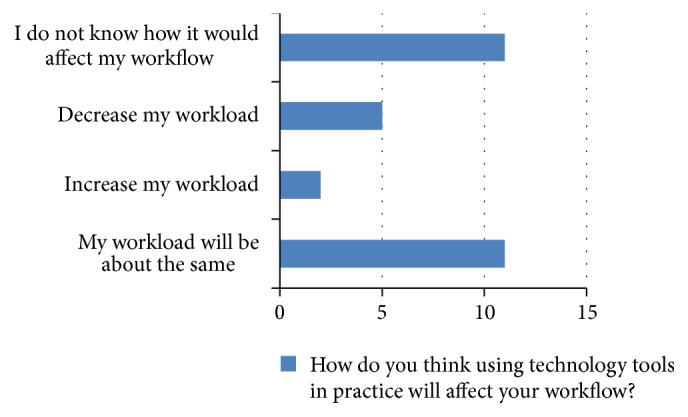
Workflow.

After all discussion points were covered, the facilitator asked for any additional comments or questions. While the group was talking, field notes were kept by the research assistant. Lastly, the participants were asked to complete a questionnaire with demographic information and their past, current, and future use of technology (Figures 1, 2, and 3 and Tables 1 and 2). Due to purposive sampling to select participants, data saturation was not the aim of data collection.

### 2.4. Data Analyses

The field notes and questionnaire responses were entered into a Microsoft access database. Then, content analysis was used to analyse the data. The researchers identified recurring themes found in the deidentified data and counted all instances of a given theme. Several procedures were employed to maximize the transcription quality and to ensure that quality standards were maintained [10]. Field notes were cross-checked by the research team members for accuracy. Multiple team members participated in the analysis of the transcriptions by identifying, discussing, and agreeing on the main points in participant responses. The results of each focus group were then summarized and are presented in Results. Some participant quotes are included to demonstrate the perspective of the participant.

## 3. Results

The demonstration of mI SMART was attended by 37 participants (group 1 *N* = 15, group 2 *N* = 12, and group 3 *N* = 10). Thirty-one participants stayed for group discussion of the mI SMART platform (group 1 *n* = 14, group 2 *n* = 11, and group 3 *n* = 6) and 29 participants, both prescribing (*N* = 8) and nonprescribing healthcare team members (*n* = 21), completed the questionnaire (group 1 *n* = 12, group 2 *n* = 12, and group 3 *n* = 5). There are 22 full time employees, an executive board of 8, and 14 volunteers from the local university. All of the participants had used technology such as EHR. However, only 2 participants had previously used technology tools, other than the EHR. Nineteen providers (65%) reported that they would definitely use mI SMART in the future.

### 3.1. Attributes

Providers identified the following attributes as what they liked best about the specific technology, mI SMART: capability to provide virtual visits, readability, connectivity of several different monitors in one system, ease of use, real-time feedback, and enhanced access. Providers in all three focus groups reported that the patient home screen was simple and easy to use. An example provider comment was “I can look at the home screen and tell what will be in each tab. I really like being able to use this on a touch screen. I think this would be simple to teach patients.” Another provider said “I think using technology would have to be made easy to use like this. I mean, I think this is easy to use.” Other providers liked that the system gave real-time feedback stating, “It's great that they get immediate feedback on their readings. It's good that they get a messages telling them if their glucose was normal or not and what to do if it's not normal.” Multiple providers agreed that mI SMART would help their patients who traveled long distances and that the technology was more patient-centered than traditional visits. “This would save so much time for my patients that drive from far away.” Lastly, the ability to use any device that connects to the internet was given as a positive attribute of mI SMART as well as the incorporation of common parts of a primary care visit integrated into one platform.

### 3.2. Barriers

Several themes related to barriers of using this type of technology in their practice emerged across all focus groups including patient ability/willingness, lack of reimbursement for services, and lack of integration into the Electronic Medical Record (EMR). The biggest obstacle that healthcare providers perceived when prescribing technology tools is patient ability or willingness to use the technology. One provider stated

*I'd really have to think about which patients would use and benefit from this technology before I would consider offering it to them. You know? I think the equipment would be a lot of money and then there is the time we would spend teaching them how to use it. Which patients would use it and which patients would be able to use it and then which patients would want to use it. *

Other providers queried use with elderly patients and patients with decreased health literacy. The majority of the providers said they would need more information on how the use of systems like this would be reimbursed prior to using it in their clinic. Another obstacle identified was the lack of integration into the existing EHR. One provider said “I do not think this will catch on in practice unless it's in the EHR. It would just be another thing I'd have to check and I already have enough to do.” In another group, a provider asked “Would you be able to see all of the patient's self-monitoring readings in the chart? If not, I'm not going to remember to look at them.”

Providers identified several potential obstacles for patients including time, alarm fatigue, ability, technical support, and having multiple chronic illnesses. Across all three focus groups, providers described time to do the required self-monitoring as the a significant barrier for using technology. An example comment was “You would really have to prioritize using this system daily. I'm not sure, if I was the patient, I would have enough time to do it all.” Other providers talked about the concept of alarm fatigue or being overwhelmed by constant reminders for monitoring. However, some liked the notifications and wondered if flexibility to set reminders based on individual preference or need was possible. Providers questioned patient ability to use the technology emerged as a theme. One provider commented “I'm not sure patients much older than me would have the ability to use all of this technology. It would take some training to teach this old dog new tricks.” This led others to ask about what would happen if there were technical glitches and the need technology support. Lastly, having multiple complex chronic illnesses was discussed in all three groups. One provider wondered aloud “Maybe this would make caring for complex chronic illness more manageable for me as a patient, or maybe it would make it more complex.” Another joined in saying

*Perhaps it would help patients realize that some of their illness are tied together… improving one, often improves the other. Like, decreasing their weight improves their blood sugar. They would be able to see all their information in front of them. *

### 3.3. Suggestions for Improvement

Adding symptom monitoring and more devices to provided home patient monitoring were the themes that emerged related to what should be added to mI SMART. The biophysical outcomes such as glucose, A1C, blood pressure, weight, lipids, INR (International Normalized Ratio), and Pulmonary Function Tests were the most frequently requested outcome measures. Providers commented that adding a box where patients could type how they feel may offer more detailed symptom monitoring and insight into how to better help them. Six providers reported wanting to see patient self-reported outcomes such as mood, symptoms, patient perceived health changes, quality of life, depression symptoms, suicidal ideation, patient satisfaction, and self-efficacy. Additionally, adding information on adherence to lifestyle behaviors was mentioned in two groups. One provider said “I'd like to know how they are sticking to their diet, exercise and medications. If they could log this, I would be able to identify what education and support was needed.” Two providers commented that the platform focuses on metabolic disorders and that they would like to see other chronic illnesses monitoring for illness like CHF and COPD. Another provider commented “It would be nice if other self-monitoring devices like their fit bit would be incorporated.” Yet another asked “Is it possible for patients to add additional information about themselves in the system like labs from another healthcare system or previous medical records?”

## 4. Discussion 

The participation of 37 individuals, both prescribing and nonprescribing, demonstrates interest in including technology into routine care of patients. No research literature can be found related to the numbers of primary care providers who are using technology other than EHRs in primary care practices. In 2014, The American Telemedicine Association reported that there were about 200 telemedicine networks in the US, with 3,500 service sites. However, the number of these in primary care is unknown. While the vast majority of participants in these focus groups have not used technology in the past, they were optimistic about mI SMART and contributed ideas about how to improve the system for both providers and patients.

The clinic of interest where the first trail of mI SMART will be conducted is a free clinic that is transitioning to Federally Qualified Health Centers (FQHC) accepting Centers for Medicare & Medicaid Services (CMS) payment. Hence, some of the providers and the executive board are concerned regarding the lack of reimbursement for services provided via electronic means. Cost effectiveness while using technology has been supported in the literature [11]. However, lack of reimbursement for technology use continues to be an obstacle. Payment and coverage for services delivered via technology is one of the biggest challenges for adoption by practices. In the United States, only 7 of 50 states have a supportive policy background that supports technology adoption [12]. The mI SMART team has been working with the clinic to determine appropriate billing procedures for offering this type of technology. Additionally, we will be completing a cost and time investment analysis during the first trial of the system.

The lack of integration into existing EHRs and capability to include personal fitness monitors and expanding self-monitoring to include symptoms, quality of life, and chronic illness other than metabolic disorders remains a challenge. We have developed mI SMART so that it is meets the standards for transferring healthcare information, HL7, into multiple health records. The last step of the first mI SMART trial will be integrating the data into the existing EHR. The system has the capability to monitor self-management of multiple chronic illnesses. However, the first trial will only address the most common chronic illnesses found in the clinic, due to cost of equipment. Additionally, the first trial of mI SMART will not include incorporation of personal fitness monitors, symptom monitoring, or a food diary. However, plans for incorporating this monitoring are being developed with the input of the clinicians and patients in this clinic. Additionally, we have added measures of quality of life, depression, loneliness, and self-management ability to the first trial in order to gain baseline data.

## 5. Conclusions 

Currently, technology innovations have emerged in silos of incompatible systems with entrenched company legacies to overcome. Future work related to technology development should consider the preferences of providers and patients, need for policy changes related to reimbursement for care, cost effectiveness of technology for patients, and future potential for integrating with existing technologies. Technology interventions have the potential to improve access and outcomes but will not be successful without the input of users. Findings from these focus groups provided essential information that informed the continued development of the mI SMART system. The demonstration of the mI SMART platform to the healthcare providers who are intended to use it was a critical step prior to feasibility testing. Once feasibility testing is complete, the next logical step will be a larger clinical trial.

## Figures and Tables

**Table 1:** Demographic information (the age ranged from 23–64 years with mean age being 41 years (SD = 10.3) and time in practice ranged from 6 months to 36 years).

Demographic	*N*	%
*Gender*		
Male	7	24.1
Female	22	75.9

*Age (range 23*–*64)*		
20–30	4	13.8
31–40	7	24.1
41–50	6	20.7
51–60	6	20.7
Over 60	6	20.7

*Practice area*		
Outpatient clinic	18	62.1
Academic/faculty practice	6	20.7
Home care	3	10.3
Hospital	2	6.9

*Time in practice (range 6 mo–36 years)*		
Less than 5 years	7	24.1
5–10 years	1	3.4
11–20 years	8	27.6
More than 20 years	13	44.8

**Table 2:** Perceptions of the mI SMART platform.

Positive attributes	Identified patient obstacles	Identified system obstacles	Suggestions for improvement
Virtual visits	Technology ability	Lack of integration	Increasing capability for biophysical monitoring
Readability	Willingness	Cost	Increasing capability for symptom monitoring
Connectivity	Time		
Ease of use			
Real-time feedback			
Enhanced access			
